# Determination of Essential Minerals and Trace Elements in Edible Sprouts from Different Botanical Families—Application of Chemometric Analysis

**DOI:** 10.3390/foods11030371

**Published:** 2022-01-27

**Authors:** Justyna Dobrowolska-Iwanek, Paweł Zagrodzki, Agnieszka Galanty, Maria Fołta, Jadwiga Kryczyk-Kozioł, Marek Szlósarczyk, Pol Salvans Rubio, Isabel Saraiva de Carvalho, Paweł Paśko

**Affiliations:** 1 Department of Food Chemistry and Nutrition, Jagiellonian University Medical College, Medyczna 9, 30-688 Kraków, Poland; justyna.dobrowolska-iwanek@uj.edu.pl (J.D.-I.); pawel.zagrodzki@uj.edu.pl (P.Z.); maria.folta@uj.edu.pl (M.F.); jadwiga.kryczyk@uj.edu.pl (J.K.-K.); 2 Department of Pharmacognosy, Jagiellonian University Medical College, Medyczna 9, 30-688 Kraków, Poland; agnieszka.galanty@uj.edu.pl; 3 Department of Inorganic and Analytical Chemistry, Jagiellonian University Medical College, Medyczna 9, 30-688 Kraków, Poland; m.szlosarczyk@uj.edu.pl; 4 Faculty of Pharmacy and Food Science, University of Barcelona, Diagonal Campus, Joan XXIII 27-31, 08-028 Barcelona, Spain; polrudu@gmail.com; 5 Mediterranean Institute for Agriculture, Environment and Development, University of Algarve, 8005-139 Faro, Portugal; icarva@ualg.pt

**Keywords:** sprouts, trace elements, essential minerals, diet

## Abstract

Background: elemental deficiency may result in the malfunctioning of human organisms. Sprouts, with their attractive looks and well-established popularity, may be considered as alternative sources of elements in the diet. Moreover, the uptake of micro- and macronutrients from sprouts is better when compared to other vegetable sources. The aim of the study was to determine and compare the level of the selected essential minerals and trace elements in 25 sprouts from different botanical families, to preselect the richest species of high importance for human diets. Methods: the Cu, Zn, Mn, Fe, Mg, Ca determinations were performed using atomic absorption spectrometry with flame atomization and iodine by the colorimetric method. Results: beetroot sprouts had the highest levels of Zn, Fe, and Mg, while onion sprouts were the richest in Mn and Ca, among all of the tested sprouts. Sprouts of the *Brassicaceae* family were generally richer in Ca, Mg, and Zn than sprouts from the *Fabaceae* family. Results allow preselection of the most perspective sprouts as possible dietary sources of essential minerals and trace elements. For rucola, leeks, onions, and beetroot sprouts, the data on minerals and trace element compositions were performed for the first time.

## 1. Introduction

Essential minerals and trace elements are crucial for the proper maintenance of the human body, due to the direct impact on its metabolic and physiological functions. The major essential minerals for humans are calcium, phosphorus, potassium, sodium, and magnesium, while iron, copper, zinc, manganese, iodine, and selenium are trace elements. The compounds have specific biochemical functions in the human body and their deficiencies may cause malfunctioning of an organism, contributing to the development of some non-communicable diseases, such as diabetes, cardiovascular diseases, or cancer [1]. Except for supplementing essential minerals and trace elements, in the form of tablets or capsules, the strategy for preventing such deficiencies includes the constant search for novel components of the human diet, rich in macro- and micronutrients. Sprouts, with their attractive looks, distinctive tastes, delicate textures, and well-established popularity among consumers, may be considered possible alternative sources of the elements. Using sprouts as food supplements is a new and interesting approach to improve the nutritional values of different by-products (i.e., breads). Fresh or lyophilized sprouts are gaining popularity, not only in daily nutrition, but also in the food industry, as a source of nutrients with antioxidant properties, which is of great importance for human health. The profiles and concentrations of these substances depend on the type and variety of the plant, agronomic conditions of growth, duration of germination, light availability and conditions, storage, and processing [2,3]. It is well documented that sprouts of different botanical origins can be good sources of proteins, sugars, fats, and dietary fibers [4]. During germination, sprouts can also accumulate and produce various bioactive compounds, such as polyphenols: flavonoids and phenolic acids [5,6,7], vitamins [8,9,10], γ-aminobutyric acid (GABA) [11], melatonin [12], D-chiro-inositol [13], or glucosinolates and their derivates [14]. Growing sprouts is a cheap and easy process that does not require sophisticated equipment and that provides high yields. All of these features make sprouts from different botanic families alternative sources of phytoconstituents and minerals, important for human health [3,15,16,17,18]. Moreover, sprouts can be grown all year round, at home, and they reach maturity in most cases within 5 to 14 days, depending on the crop nature and botanical origin [16]. Due to the fact that sprouts are harvested at such an early stage of plant growth, and are intended for eating as a whole, with the seeds, they are the richest in nutrients and phytochemicals, compared to plants at the later stages of development [2].

Sprouts seem to also be important sources of essential minerals and trace elements, characterized by high availability for human organisms. During sprouting time, the level of compounds that can negatively influence the availability of essential and trace elements from vegetables (e.g., indigestible dietary fiber, phytates, oxalates, and tannic acids) decrease significantly [4,19,20,21]. They are commonly present in foods derived from cereals or legumes and may form insoluble complexes with minerals, such as potassium, iron, zinc, calcium, magnesium, copper, and manganese [16,22]. In particular, phytic acid is considered as an important factor limiting the possible use of many plant-based food products as sources of these elements for living organism. Phytates decrease the absorption of the minerals in monogastric animals, while in humans, they can lead to mineral deficiencies, due to the lack of phytase, the enzyme that hydrolyzes phytates and releases the bound elements [23]. During the sprout germination process, phytates are degraded by intrinsic phytases and used as sources of inorganic phosphate by plant seeds, likely resulting in an increase of the nutritional impacts of the germinated seeds [22]. It was observed that the activity of phytases significantly increased during the sprouting time, even up to eight-fold [4], resulting in the improvement of the bioavailability of the minerals, as was shown for iron and calcium in germinated millet, green grass, cowpeas, lentils, and chickpeas [24,25].

The bioavailability of minerals depends on the chemical form or species present in by-products. Conversion of the applied inorganic salts into organically bound or chelated species can occur during the germination [26]. For most essential and trace elements, inorganic salts are only slightly absorbed by the human body, whereas the uptake of organically bound or chelated trace elements from food is considerably higher, e.g., the intestinal absorption of Fe, Zn, Mn, Ca, Mg, and Cu is greater when they are ingested as amino acid chelates rather than as inorganic salts [26,27]. It was especially confirmed for selenium, which, as selenite, selenate, and Se nanoparticles taken in by roots and sprouts, were prone to convert to organic forms of Se, including selenomethionine, selenocysteine, and MeSeCys, which are beneficial to plants as well as for humans, as it is better absorbed in the intestine [28,29,30]. It should also be pointed out that, unlike the other plant food products, where negatively charged functional groups of phytochemicals can bind positively charged di- and trivalent cations, resulting in limited bioavailability of macro- and micronutrients, the elements present in sprouts may be converted to chelates by complexing reactions with organic acids (citric acid), which increases their bioavailability [26]. All of these arguments support the use of sprouts as alternative dietary sources of the elements.

Many plant species deliver their seeds for germination and sprout production, with legumes (e.g., soybean, lentil, pea); cereals (e.g., barley, oat, wheat), and pseudocereals (e.g., buckwheat, amaranth, quinoa); oilseeds (e.g., sunflower, hazelnut, sesame); or some vegetables (e.g., cabbage, broccoli, clover) being the most popular [31]. Referring to their botanical classification, these most popular sprouts belong to *Brassicaceae* (rutabaga, broccoli, kale), *Fabaceae* (soia, chickpea, beans), *Amaryllidaceae* (onion, garlic, leek), *Poaceae* (wheat, barley, oat), *Amaranthaceae* (beetroot, amaranth), or *Asteraceae* (sunflower) families. Depending on the botanical origin, the sprouts differ in their chemical compositions, which translates into a variety of their tastes, as well as different times of reaching consumption maturity [32]. However, information on the compositions of essential minerals and trace elements in the sprouts are scarce, and barely concern a few plant species, such as wheat, buckwheat, and quinoa [26], pea [33], cabbages [34] or Adzuki bean [35]. Moreover, the results of the studies were obtained using different analytical methods, which makes the comparison of the elemental content difficult. Thus, the aim of the study was to determine and directly compare the levels of the selected essential minerals (calcium, magnesium) and trace elements (iron, zinc, manganese, copper, and iodine) from 25 kinds of edible sprouts from different botanical families, to preselect the richest species of high importance for the human diet.

## 2. Materials and Methods

### 2.1. Chemicals

Suprapur^®^ 65% (*w*/*v*) nitric acid and Ultrapur^®^ 30% (*w*/*v*) hydrochloric acid were purchased from Merck (Darmstadt, Germany) and Fluka (Steinheim, Germany), respectively. One elementary standard stock solution of Cu, Zn, Mn, Fe, Ca, Mg (ultra-grade, 1000 mg/L) was supplied by Merck (Darmstadt, Germany). TraceSELECT™ cesium chloride–lanthanum chloride buffer solution was obtained from Fluka (Buchs, Switzerland). Analytical grade potassium iodate, ammonium persulfate, 98% sulfuric acid, sodium chloride were purchased from ChemPUR (Piekary Śląskie, Poland). ReagentPlus^®^ arsenic (III) oxide, ammonium cerium (IV) sulfate dehydrate (ACS grade) were from Sigma-Aldrich (Seelze, Germany). Ultrapure water of 18 MΩ cm was obtained from Milli-Q Direct-Q^®^ 3 UV Water Purification (Merck-Millipore, Burlington, MA, USA).

### 2.2. Sprouts

A total of 25 kinds of edible sprouts were evaluated in the study: 8 kinds of sprouts from *Fabaceae* family: mung (*Vigna radiata*), lentil (*Lens culinaris*), lucerne (Alfalfa) (*Medicago sativa*), pea (*Pisum sativum*), soy (*Glycine max*), Adzuki beans (*Vigna angularis*), kidney beans (red) (*Phaseolus vulgaris*) and fenugreek (*Trigonella foenum-graecum*); 11 kinds of sprouts from *Brassicaceae* family: rutabaga (*Brassica napus* var. *napobrassica*), radish (*Raphanus raphanistrum* subsp. *sativus*), broccoli (*Brassica oleracea* var. *italica*), kale (*Brassica oleracea* var. *sabellica*), red kale (*Brassica oleracea* var. *acephala*), red cabbage (*Brassica oleracea* var. *capitata*), rucola (*Eruca sativa*), China rose (*Raphanus sativus*), white mustard (*Sinapis alba*), kohlrabi (*Brassica oleracea* convar. *acephala* var. *gongylodes*), bittercress (*Cardamine impatiens*); 2 kinds of sprouts from *Amaryllidaceae* family: leek (*Allium porrum*), onion (*Allium cepa*); 2 kinds of sprouts from *Poaceae* family: barley (*Hordeum vulgare*), wheat (*Triticum aestivum* ssp. *vulgare*), one sprouts from *Amaranthaceae* family: beetroot (*Beta vulgaris*), and 1 sprout from *Asteraceae* family: sunflower (*Helianthus annuus*). The seeds for sprouts were bought from different companies, available for consumers in Poland.

Before seeding, the seeds were immersed in distilled water for 3 h, to obtain stable conditions during the harvesting process. Then the seeds were grown for 7 days after seeding in EQMM EasyGreen MikroFarm at a temperature of 23 ± 2 °C, in natural light conditions, and were watered with distilled water 3 times a day. After harvesting, the sprouts were freeze-dried, as described previously [3].

### 2.3. Mineralization of Sprout Samples for Multi-Element Analysis

Samples of the freeze-dried sprouts were mineralized according to the procedure described previously [36]. Briefly, 0.5 g of the freeze-dried sprouts was transferred into a high-pressure Teflon vessel and mixed with 7 mL of concentrated nitric acid. The digestion process was carried out in the microwave oven MDS 2000 (CEM, Matthews, NC, USA). After the mineralization process was complete, the vessels were cooled to room temperature and purged with compressed air for 10 min to remove the interference components. Finally, 6 M HCl was added to the mineralizates. For validation of the digestion and determination methods, two standard reference materials (NISTSRM 1575 pine needles and BCR SRM 62 olive leaves), representing similar matrices, were analyzed simultaneously.

### 2.4. Determination of Selected Elements by Flame AAS

The determination of Cu, Zn, Mn, Fe, Mg, Ca was performed using atomic absorption spectrometry (Perking Elmer 5100, Waltham, MA, USA) with flame atomization (FA). For quantification of metals, the calibration curves were prepared with standard solutions of 0, 1.25, 2.5, 5, 10 and 20 mg/L for Ca and Mg; 0, 0.125, 0.25, 0.5, 1.0, 2.0, and 3.0 mg/L for Cu and Zn; 0, 0.5, 1.0, 2.0, 3.0, and 5.0 mg/L for Fe. Buffer modifiers, such as CsCl and LaCl_3_, were used to avoid chemical interference (Ca and Mg measurement). To determine the manganese concentration in sprouts, the standard addition method was used. For this purpose, for each type of sprout, 4 mineralized solutions (0.5 mL each) were prepared. Then the samples were completed with 1 mL of manganese standard solutions of 0.5, 1, and 2 mg/L, while 1 mL of 0.5% HNO_3_ was added to the fourth sample (blank sample). The results are shown as mg or mg/100 g of dry matter (DM).

### 2.5. Determination of Iodine in Sprouts

Iodine compounds were extracted from sprouts according to the procedure described by Paśko et al. [37]. A total of 10 g of fresh sprouts was immersed in 90 mL of ultrapure water and exposed to ultrasounds. The samples were then centrifuged, and the supernatant was taken for further analysis. Iodine in the water sprout extracts was determined by a modified colorimetric method of Sandell–Kolthoff after ammonium persulfate digestion [37,38]. The principle of this method relies on the iodine catalyzed redox reaction between cerium (IV) (yellow) and arsenic (III), to cerium (III) (colorless) and arsenic (V). Measurements were made at a wavelength of 420 nm—maximum cerium (IV) absorbance. The results are shown as mg/100 g of DM.

### 2.6. Statistical Approach

Descriptive statistics were calculated for all obtained parameters. The comparison between the groups of sprouts was performed using either t-Student or Mann–Whitney tests (when appropriate), which were applied only to those groups of sprouts with sizes equal or bigger than 5 (i.e., *Fabaceae* and *Brassicaceae*). Differences with *p* < 0.05 were considered statistically significant. Principal component analysis (PCA) was applied to describe the correlation structure between concentrations of elements determined in sprouts. For more detailed insights, the analysis of component weights in the PCA model was conducted. The description of our approach to the PCA model was provided in a number of previous papers [39,40]. Statistical analyses were performed using the packages: Statistica v. 13.3. (TIBCO Software, Inc., Palo Alto, CA, USA) and SIMCA-P v.9 (Umetrics, Umeå, Sweden). The correlation weights were calculated using software delivered by MP System Co. (Chrzanów, Poland).

## 3. Results and Discussion

In our study, we searched for a preselection of the novel, rich dietary sources of essential minerals and trace elements among the sprouts of different botanical origins, which can be considered as complementary reservoirs of the nutrients. The key for the choice of the 25 plant species and varieties, used in the study, was their presence on the market and popularity among the consumers, but, on the other hand, some less popular and underestimated species were also included in the study. The species chosen for the study belonged mainly to the *Fabaceae* (8) and *Brassicaceae* (11) families, according to their popularity, while the other families, namely *Amaryllidaceae, Poaceae, Amaranthaceae,* and *Asteraceae*, were represented by one or two species. Such a wide comparison of the species and varieties of sprout-giving plants was performed for the first time. All of the tested sprouts were provided with the same temperature, light, and water growing conditions, to enable further comparisons between the species. The determination of the content of the examined elements was performed by the standard AAS method, excluding iodine, which content was determined by the colorimetric method. The concentrations of essential minerals and trace elements in the tested sprouts are presented in Table 1.

**Figure 1:**
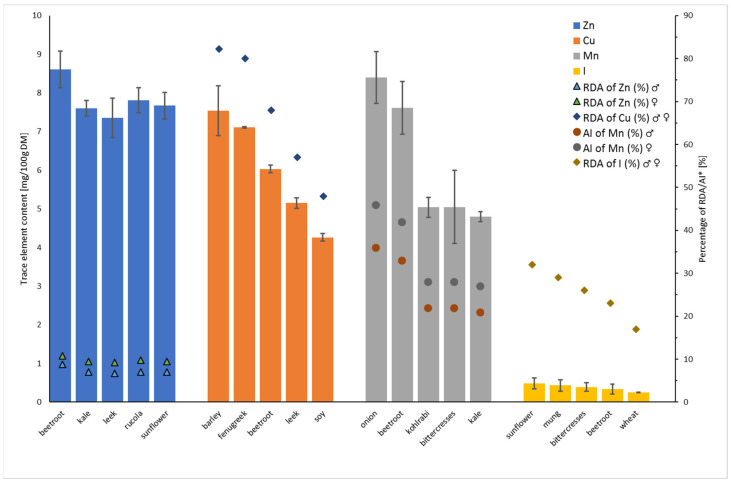
The concentrations of trace elements (Zn, Cu, Mn, I) in the sprouts from the top five groups (see explanation in the text above), with the reference to % of RDA or AI* through consumption of 100 g of fresh sprouts. *% of Recommended daily allowance (RDA) and adequate intake (AI) based on human nutrition standards for the Polish population [41]. RDA for Zn for males (31–50 years) is 11 mg/day/person and for females (31–50 years) is 8 mg/day/person; RDA for Cu for males (31–50 years) and females (31–50 years) is 0.9 mg/day/person; RDA for I for males (31–50 years) and females (31–50 years) is 0.15 mg/day/person; AI for Mn for males (31–50 years) is 2.3 mg/day/person and for females (31–50 years) is 1.8 mg/day/person.

**Figure 2:**
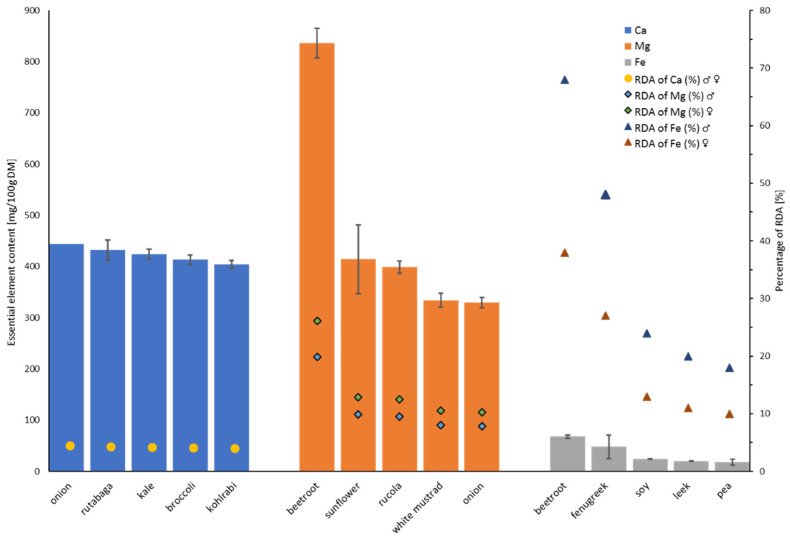
The concentration of essential minerals and iron in the sprouts from the top five groups (see explanation in the text above), with the reference to % of RDA* through consumption of 100 g of fresh sprouts. *% of Recommended daily allowance (RDA) based on human nutrition standards for the Polish population [41]. RDA for Ca for males (31–50 years) and females (31–50 years) is 1000 mg/day/person; RDA for Mg for males (31–50 years) is 420 mg/day/person and for females (31–50 years) is 320 mg/day/person; RDA for Fe for males (31–50 years) is 10 mg/day/person and for females (31–50 years) is 18 mg/day/person.

Additionally, for each evaluated element, the richest top five sprouts were chosen, to enable the preselection of the most promising species, and the results are presented in Figure 1 for selected trace elements, and Figure 2 for essential minerals and iron, with the references for recommended daily allowance (RDA) or adequate intake (AI) of fresh sprout consumption.

### 3.1. Concentration of Essential Minerals in Sprouts

The highest concentration of magnesium was found in beetroot sprouts (836 ± 29 mg/100 g DM), which refers to almost 20% and 26% of the daily requirements for males and females, respectively (Figure 2), while the lowest in lentil (82 ± 4 mg/100 g DM) and barley (94 ± 3 mg/100 g DM) sprouts. As almost no data exist on the content of magnesium in the tested sprouts, our results can only be compared to those by Santos et al. [42] for 12 lentil varieties. The obtained concentration of magnesium in 5-day-old sprouts ranged from 89.1 to 124.7 mg/100 g DM, which is higher than in our experiment for 7-day sprouts. The determined calcium content in the examined sprouts were very diversified. The highest amount of calcium was detected in onion (444 ± 22 mg/100 g DM), rutabaga (432 ± 18 mg/100 g DM), and kale (424 ± 11 mg/100 g DM) sprouts, with the lowest level for wheat sprouts (18 ± 1 mg/100 g DM). Interestingly, even the highest obtained results cover only about 4% of the daily requirements of calcium (Figure 2). Our results for mung sprouts are comparable to those obtained by Nunes et al. [43] in the 5-day-old sprouts (120 mg/100 g DM), Machado et al. [44] and El-Adawy et al. [45], with calcium levels varied from 121 mg/100 g DM in seeds to 140 mg/100 g DM in 6-day-old mung sprouts, respectively. The fenugreek sprouts analyzed by Pająk et al. [20] had lower calcium concentrations (1.09 ± 0.09 mg/100 g DM) when compared to those in our study. In the case of Adzuki sprouts, the calcium content noted by Nunes et al. [43] was significantly higher (360 mg/100 g DM) in comparison to our results.

### 3.2. Concentration of Trace Elements in Sprouts

The concentration of iron in the examined sprouts ranged from 5.2 ± 0.1 mg/100 g DM (mung sprouts) to 68.0 ± 3.3 mg/100 g DM (beetroot sprouts), and the latter result covers almost 70 and 40% of the daily requirements for males and females, respectively (Figure 2). Machado et al. [44] found an increase in iron concentration during the germination of mung sprouts (from 4.35 for seeds to 5.58 mg/100 g DM for 6-day-old sprouts). The iron concentration determined in our study was comparable to the values obtained by Machado et al. [44], but lower than that determined by Nunes et al. (9.25 mg/100 g DM) [43].

Among all of the tested sprouts, beetroot sprouts (8.61 ± 0.47 mg/100 g DM) had the highest concentration of zinc, and in wheat sprouts (2.12 ± 0.07 mg/100 g DM) the level was the lowest. The zinc content in the sprouts with the highest results did not exceed 10% of the daily requirements for this element (Figure 1). The mean concentrations of zinc (2.95 mg/100 g DM), determined by the Lintschinger et al. [26] in 3-day-old wheat sprouts were higher than those determined in wheat sprouts in our study. The average concentration of zinc in the tested sprouts from the *Fabaceae* family ranged from 3.13 ± 0.03 (red beans) to 6.25 ± 0.49 mg/100 g DM (alfalfa). Our results agree with Mun et al. [46] for three cultivars of red bean 7-day sprouts (2.88 ± 0.03–6.32 ± 0.03 mg/100 g DM). Zinc content in 5-day-old pea sprouts, observed by Bączek-Kwinta et al. [47], was comparable (4.54 ± 0.17 mg/100 g DM) to our study, while the study of Zou et al. [48] for 5-day-old soybean sprouts indicated a lower concentration of zinc (3.2 ± 0.1 mg/100 g DM) in comparison to our study. The content of zinc in 4-day Adzuki, soybean, and mung bean sprouts (2.50 ± 0.06, 4.66 ± 0.09, 2.70 ± 0.08 mg/100 g DM, respectively) was lower than that obtained in our experiment [19], while for lentil and fenugreek (3.49 ± 0.35 mg/100 g DM) sprouts, the zinc level was of comparable value to our results [20]. In the case of copper, its highest concentration was observed in barley (7.54 ± 0.64 mg/100 g DM) and fenugreek sprouts (7.11 ± 0.02 mg/100 g DM), with the lowest result for mung bean (1.24 ± 0.05 mg/100 g DM) and rutabaga (0.37 ± 0.01 mg/100 g DM) sprouts. The content of copper in the sprouts from our top five group covers from 47 to 80% of the daily requirements (Figure 1), which is the best result obtained among all the determined elements. Pająk et al. [20] observed a much lower concentration of this element in 7-day-old fenugreek sprouts at the level of 1.09 ± 0.09 (mg/100 g DM). Swieca et al. [19], analyzing selected sprouts from the *Fabaceae* family, obtained lower mean concentration values for lentil (0.77 ± 0.02 mg/100 g DM), Adzuki bean (0.70 ± 0.01 mg/100 g DM), soybean (1.13 ± 0.02 mg/100 g DM), but comparable in case of mung bean (1.12 ± 0.10 mg/100 g DM) sprouts, than those obtained by in our study. Zou et al. [48] noted a similar level of copper (1.16 ± 0.03 mg/100 g DM). The results obtained by Mun et al. [46] for copper level in three cultivars of red bean sprouts, with mean values ranging from 0.73 ± 0.0 to 1.16 ± 0.0 mg/100 g DM, are in agreement with our study. The average concentration of copper in alfalfa sprouts determined in our study was comparable to the range 1.71 ± 0.07 mg/100 g DM—2.01 ± 0.23 mg/100 g DM, obtained by Chiriac et al. [49]. The highest manganese content was found in onion sprouts (8.40 ± 0.67 mg/100 g DM), which covers almost 40 and 50% of the daily requirements for males and females, respectively (Figure 1), and the lowest in barley sprouts (1.53 ± 0.18 mg/100 g DM). Fazaeli et al. [50] monitored the concentration of eight elements in samples of barley sprouts between the sixth and eighth day of germination in a still hydroponic growing chamber. The results indicated that the concentration of copper and manganese changed with plant growth from 5.35 to 7.23 mg/kg DM and 20.3 to 17.8 mg/kg DM, respectively. The manganese concentration (1.53 ± 0.18 mg/100 g DM) determined in our study was similar, and the copper concentration was an order of magnitude higher than that determined by Fazaeli et al. [50] in barley sprouts on the eighth day of germination. What should be noted, our study is probably the first to determine manganese content in onion sprouts. In the case of iodine content in the analyzed sprouts, the concentration did not exceed the value of 0.5 mg/100 g DM, with the highest result for mung bean sprouts (0.43 ± 0.15 mg/100 g DM) and sunflower (0.48 ± 0.14 mg/100 g DM) and the lowest in red bean (0.03± 0.00 mg/100 g DM), pea bean (0.03 ± 0.01 mg/100 g DM), and Adzuki bean sprouts (0.04 ± 0.02 mg/100 g DM). However, it is worth to underline that the content of iodine in the tested sprouts covers from 17 to 32% of the daily requirements for this element (Figure 1). The published studies, to date, concerning the levels of iodine in the sprouts of various types of plants, are scarce. Jerše et al. [33] examined iodine content in 14-day-old pea sprouts and the results were an order of magnitude higher when compared to our study, while the concentration of iodine in 4-day rutabaga sprouts (about 0.065 mg/100 g DM), noted in our previous study [37], was lower than that obtained during this study.

### 3.3. Chemometric Analysis

**Figure 3:**
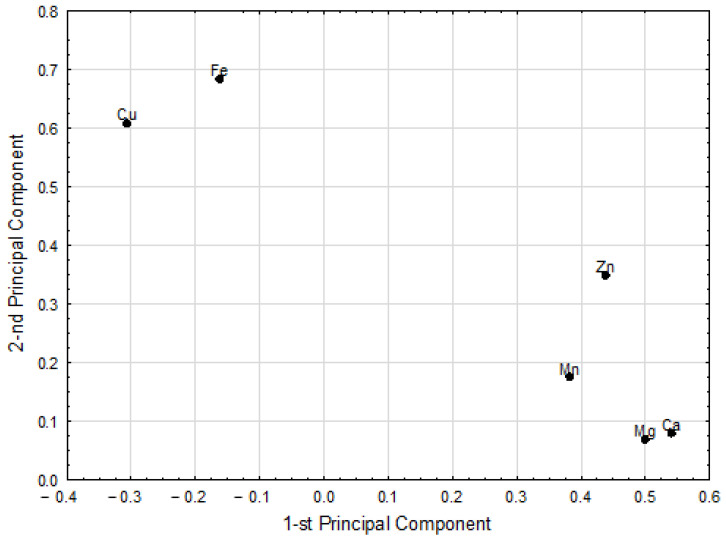
The weights of the first two principal components of the PCA model.

The PCA model fulfilling cross-validation criteria was constructed, to highlight the possible similarities in elemental pattern within the tested sprouts. The model had two significant principal components, with eigenvalues of 2.84 and 1.69, respectively, which explained 75.5% of the variance of studied parameters. The iodine concentration was not included in the model as noninformative (i.e., not significantly correlated with other parameters). The sample of beetroot sprouts was excluded from the PCA model as an outlying object. The loadings for the first two principal components are shown in Figure 3.

The first principal component in this model had positive weights predominantly for the Ca and Mg. Consequently, the highest positive correlation weights based on this component were revealed between these two elements. Both were correlated with Zn and Mn (Figure 3, Table 2). The second principal component was loaded mainly by Fe and Cu (Figure 3). Therefore, these two elements had a very high correlation weight (Table 2). Among the relatively strong correlations, there were also correlations between Zn and two groups of elements—Fe and Cu as well Ca and Mg (Figure 3, Table 2). Teng et al. [15] described positive correlations (Fe and Ca as well Fe and Mg) of kohlrabi and broccoli microgreens.

**Figure 4:**
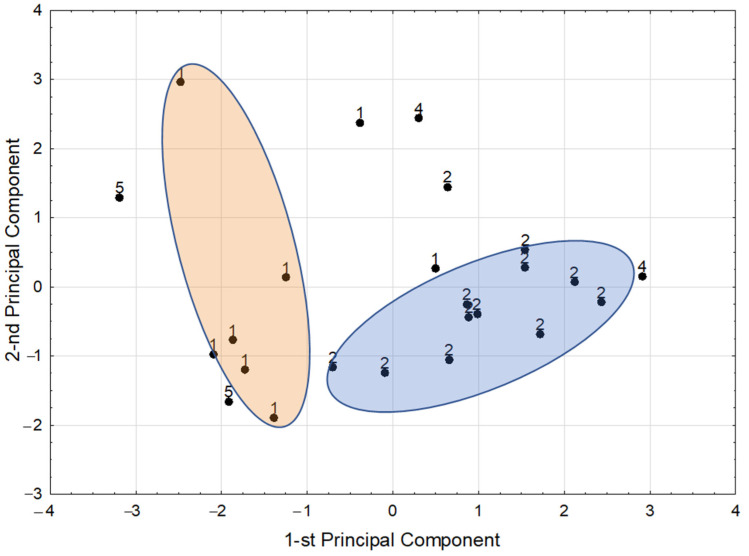
The score scatterplot of PCA model: 1—mung, lentil, lucerne, pea, soy, Adzuki beans, kidney beans (red) and fenugreek; 2—rutabaga, radish, kale, kohlrabi, bittercress; 3—beetroot (out of the model); 4—leek, onion; 5—barley, wheat.

The score scatterplot of the PCA model is shown on Figure 4. Visual inspection of this plot disclosed two homogenous clusters containing the majority of the samples from *Fabaceae* (points denoted as 1) and *Brassicaceae* (points denoted as 2) families, with the exception of alfalfa and soy from the former and bittercress from the latter family. Samples belonging to “*Brassicaceae* cluster” were in general significantly richer in Ca, Mg, and Zn than samples from the “*Fabaceae* cluster” (Ca: 354.1 ± 100.1 mg/100 g DM vs. 84.3 ± 30.8 mg/100 g DM, Mg: 291.1 ± 74.1 mg/100 g DM vs. 127.1 ± 26.2 mg/100 g DM, and Zn: 5.61 ± 1.61 mg/100 g DM vs. 3.86 ± 0.76 mg/100 g DM, respectively). The samples outside both clusters had higher Cu and Fe concentrations than samples from “*Brassicaceae* cluster” (Cu: 3.51 ± 2.48 mg/100 g DM vs. 0.89 ± 0.54 mg/100 g DM, and Fe: 15.6 ± 5.4 mg/100 g DM vs. 10.8 ± 2.4 mg/100 g DM, respectively).

The disclosed clusters were somehow expected, based on some similarities occurring within the botanical families, but in our study, such relationships were proven for the first time. What is interesting, the species from other families included in the study did not form clusters. This may be due to the small number of representatives in *Amaryllidaceae* and *Poaceae* families, but the long distance between the points representing the species of these families on the plot may suggest lower similarities between them; however, this observation needs further studies.

## 4. Conclusions

This is the first such complex and cross-sectional analysis of sprouts from different botanical families, as the potential source of essential minerals and trace elements. Our top five ranking of the sprouts indicated beetroot sprouts as the unquestionable winner, present in six out of seven “categories” of the evaluated essential minerals and trace elements, with the highest level of zinc, iron, and magnesium among all of the tested sprouts. The sprouts of kale, leek, onion, and sunflower were present in three out of seven “categories”, with onion sprouts being the richest in manganese and calcium among all of the tested sprouts. Surprisingly, the most commonly available on the market and popular sprouts of broccoli, radish, or soy were not so rich in the evaluated elements, when compared to the underestimated beetroot, onion, or leek. The results of our analysis enable the preselection of the most perspective sprouts for further processing, which may aspire to the group of functional foods and serve as an additional dietary source of essential minerals and trace elements. For most of the examined sprouts (e.g., rucola, leek, onion, beetroot) the data on their elemental compositions were performed for the first time. Thus, the results obtained during this research may significantly contribute toward broadening the knowledge on the content of the selected essential minerals and trace elements in edible sprouts, in terms of their use as the sources of these compounds.

## Figures and Tables

**Table 1:** Concentrations of essential minerals and trace elements in sprouts from different botanical families (mg/100 g DM; n = 3, mean ± SD).

	Ca	Fe	Mg	Cu	I	Mn	Zn
Sprouts	*Fabaceae*						
Mung	115 ± 3	5.2 ± 0.1	119 ± 6	1.24 ± 0.05	0.43 ± 0.15	1.86 ± 0.12	3.69 ± 0.02
Lentil	60 ± 5	12.0 ± 0.6	82 ± 4	1.55 ± 0.56	0.07 ± 0.02	1.75 ± 0.37	3.58 ± 0.35
Lucerne	351 ± 10	14.3 ± 1.0	190 ± 3	1.46 ± 0.01	0.24 ± 0.03	3.18 ± 0.14	6.25 ± 0.49
Pea	65 ± 6	17.7 ± 5.6	127 ± 12	3.12 ± 1.03	0.03 ± 0.01	3.31 ± 1.03	5.27 ± 0.26
Soy	202 ± 6	24.1 ± 0.9	233 ± 11	4.26 ± 0.10	0.12 ± 0.06	4.24 ± 0.67	5.81 ± 0.13
Adzuki beans	65 ± 2	12.8 ± 0.4	140 ± 6	1.87 ± 0.04	0.04 ± 0.02	1.82 ± 0.25	3.43 ± 0.14
Kidney beans (red)	68 ± 2	10.1 ± 0.5	133 ± 3	1.67 ± 0.08	0.03 ± 0.00	2.38 ± 0.45	3.13 ± 0.03
Fenugreek	132 ± 4	48.2 ± 23.1	161 ± 11	7.11 ± 0.02	0.18 ± 0.06	2.30 ± 0.82	4.09 ± 0.20
	*Brassicaceae*						
Rutabaga	432 ± 18	9.3 ± 0.7	312 ± 1	0.37 ± 0.01	0.10 ± 0.02	4.11 ± 0.12	5.36 ± 0.04
Radish	196 ± 6	9.3 ± 0.1	280 ± 12	0.47 ± 0.02	0.14 ± 0.02	1.71 ± 0.09	4.58 ± 0.06
Red kale	415 ± 11	9.5 ± 0.6	263 ± 4	0.62 ± 0.09	0.23 ± 0.03	2.47 ± 0.07	4.25 ± 0.40
Broccoli	413 ± 8	14.3 ± 0.5	295 ± 13	0.49 ± 0.05	0.17 ± 0.05	2.02 ± 0.23	5.32 ± 0.02
Red cabbage	392 ± 6	11.8 ± 0.4	210 ± 5	0.58 ± 0.01	0.17 ± 0.03	4.27 ± 0.24	5.02 ± 0.25
Rucola	332 ± 16	7.1 ± 0.3	399 ± 12	2.31 ± 0.06	0.21 ± 0.08	3.51 ± 0.29	7.81 ± 0.33
China rose	127 ± 8	10.7 ± 0.3	270 ± 17	0.92 ± 0.03	0.14 ± 0.03	2.09 ± 0.51	3.47 ± 0.22
White mustard	383 ± 15	15.0 ± 0.2	334 ± 14	1.22 ± 0.18	0.08 ± 0.01	3.22 ± 0.35	6.88 ± 0.07
Kale	424 ± 11	11.9 ± 0.2	163 ± 6	0.92 ± 0.13	0.24 ± 0.06	4.80 ± 0.13	7.60 ± 0.02
Kohlrabi	404 ± 17	11.9 ± 0.1	261 ± 3	1.10 ± 0.10	0.22 ± 0.05	5.04 ± 0.26	3.84 ± 0.16
Bittercress	228 ± 11	15.9 ± 0.5	314 ± 13	4.17 ± 0.10	0.39 ± 0.11	5.05 ± 0.95	5.96 ± 0.66
	*Amaryllidaceae*						
Leek	335 ± 16	20.1 ± 0.7	222 ± 6	5.15 ± 0.14	0.12 ± 0.04	3.70 ± 0.72	7.36 ± 0.51
Onion	444 ± 22	10.4 ± 0.5	329 ± 10	0.77 ± 0.03	0.10 ± 0.03	8.40 ± 0.67	5.73 ± 0.23
	*Poaceae*						
Barley	25 ± 1	15.9 ± 0.5	94 ± 3	7.54 ± 0.64	0.20 ± 0.07	1.53 ± 0.18	3.71 ± 0.25
Wheat	18 ± 1	8.5 ± 0.1	102 ± 3	1.22 ± 0.10	0.25 ± 0.01	3.61 ± 0.62	2.12 ± 0.07
	*Amaranthaceae*						
Beetroot	370 ± 10	68.0 ± 3.3	836 ± 29	6.03 ± 0.07	0.34 ± 0.13	7.61 ± 0.68	8.61 ± 0.47
	*Asteraceae*						
Sunflower	376 ± 61	11.4 ± 3.3	416 ± 47	0.82 ± 0.17	0.48 ± 0.14	3.25 ± 0.47	7.67 ± 0.34
*Fabaceae* vs. *Brassicaceae* (Mean ± SD, significance level)	132.3 ± 101.0 340.5 ± 106.7 *p* = 0.000	18.1 ± 13.4 11.5 ± 2.7 NS	148.1 ± 46.4 281.9 ± 62.3 *p* = 0.000	2.79 ± 2.03 1.20 ± 1.13 *p* = 0.043	0.14 ± 0.14 0.19 ± 0.08 NS	2.61 ± 0.89 3.48 ± 1.26 NS	4.41 ± 1.19 5.46 ± 1.46 NS

**Table 2:** Correlation weights based on principal components in the PCA model (only correlation weights with absolute values higher than 0.100 are shown).

Pairs of Correlated Parameters		Correlation Weights
Cu	Fe	0.404
Ca	Mg	0.270
Zn	Ca	0.205
Mn	Ca	0.198
Zn	Cu	0.192
Zn	Mg	0.188
Zn	Fe	0.187
Mn	Mg	0.183
Zn	Mn	0.162

## Data Availability

Data available upon request.
